# Comprehensive analysis of aberrantly expressed lncRNAs and construction of ceRNA network in gastric cancer

**DOI:** 10.18632/oncotarget.24841

**Published:** 2018-04-06

**Authors:** Kanagaraj Arun, Ganesan Arunkumar, Duraisamy Bennet, Servarayan Murugesan Chandramohan, Avaniyapuram Kannan Murugan, Arasambattu Kannan Munirajan

**Affiliations:** ^1^ Department of Genetics, Dr. ALM PG Institute of Basic Medical Sciences, University of Madras, Taramani Campus, Chennai – 600 113, India; ^2^ Institute of Surgical Gastroenterology, Rajiv Gandhi Government General Hospital and Madras Medical College, Chennai – 600 001, India; ^3^ Department of Molecular Oncology, King Faisal Specialist Hospital and Research Centre, Riyadh-11211, Saudi Arabia

**Keywords:** gastric cancer, lncRNA, ceRNA, competing endogenous RNA, miRNA

## Abstract

Gastric cancer remains fifth most common cancer often diagnosed at an advanced stage and is the second leading cause of cancer-related death worldwide. Long non-coding RNAs (lncRNAs) involved in various cellular pathways are essential for tumor occurrence and progression and they have high potential to promote or suppress the expression of many genes. In this study, we profiled 19 selected cancer-associated lncRNAs in thirty gastric adenocarcinomas and matching normal tissues by qRT-PCR. Our results showed that most of the lncRNAs were significantly upregulated (12/19). Further, we performed bioinformatic screening of miRNAs that share common miRNA response elements (MREs) with lncRNAs and their downstream mRNA targets. The prediction identified three microRNAs (miR-21, miR-145 and miR-148a) and five gastric cancer-specific target genes (EGFR, KLF4, DNMT1 and AGO4) which also showed strong correlation with lncRNAs in regression analysis. Finally, we constructed an integrated lncRNA-miRNA-mRNA interaction network of the candidate genes to understand the post-transcriptional gene regulation. The ceRNA network analysis revealed that the differentially regulated miR-21 and miR-148a were playing as central candidates coordinating sponging activity of the lncRNAs analyzed (H19, TUG1 and MALAT1) in this study and the overexpression of H19 and miR-21 could be a signature event of gastric tumorigenesis that could serve as prognostic indicators and therapeutic targets.

## INTRODUCTION

Gastric cancer is the fifth most common cancer and remains as one of the serious global health issues. As per the GLOBOCAN 2012 update, gastric cancer constitutes 6.8% of the total cancer incidences worldwide. The incidence of gastric cancer varies geographically and nearly half of the global incidence is observed in Asia especially in China, Japan, South Korea and Taiwan [1]. More than 95% cases are gastric adenocarcinoma arises at glandular epithelium of stomach due to a wide spectrum of etiological traits like ageing, *Helicobacter pylori* infection, tobacco chewing, consumption of alcohol, the habit of eating cured meat and salted food [2]. In addition, the genetic makeup of an individual is thought to be an independent and important risk factor for the gastric malignancies. Advances in next-generation sequencing and whole transcriptome analysis revealed a wide array of coding and non-coding genes that are frequently involved in gastric cancer and are often linked with different clinical stages and pathological grades [3].

Non-coding RNAs are the largest class of transcripts which constitute ~80-90% of a human transcriptome. In the recent decade, experimental evidence demonstrates that non-coding RNAs play a role in major cellular events such as transcriptional regulation, chromatin modification, and maintenance of genome integrity [4]. Long non-coding RNAs (lncRNAs) are >200 nt sized transcripts which are not translated into proteins. LncRNAs are functionally diverse molecules and their aberrant expression signatures have been widely recognized to be involved in various tumorigenic processes like proliferation, metastasis, and anti-apoptosis. Several studies reported that alteration in the expression level of these lncRNAs could modify cellular characteristics and result in gastric cancer tumorigenesis [5]. LncRNAs have been shown to have microRNA responsible elements (MRE), the binding site for microRNAs (miRNAs) by which they can potentially sponge the miRNAs that results in impaired miRNA-mediated post-transcriptional regulation of target mRNAs. In 2011, Poliseno *et al*. collectively named these transcripts as competitive endogenous RNA (ceRNA) after the experimental elucidation of interaction between PTEN and its pseudogene PTENP1 and both compete for binding with miR-21 [6]. Recently, various reports have clarified the existence of ceRNA network in multiple cancers including gastric cancer and the lncRNA FER1L4 reported to sponge miR-106a-5p by sharing common MREs between the RB1 mRNA. This regulatory interaction between FER1L4 and miR-106-5p could facilitate the escape of RB1 from miRNA-mediated regulation and its upregulation [7]. Identification of gastric cancer associated ceRNA network could be useful to understand their role in tumorigenesis and therapeutic outcome in gastric cancer.

In this study, we profiled 19 selected cancer-associated lncRNAs based on several functional studies (H19, PTENP1-AS, GAS5, MEG3, TUG1, AP5M1, PANDA, MALAT1, CCAT1, LINC312, NBAT1, HOTAIR, ZEB2-AS1, BC032469, POU3F3, UCA1, NEAT1, FALEC, LINCROR) in gastric tumors and adjacent normal tissue samples. We performed literature survey and bioinformatic analysis to predict miRNAs and their target genes that might be regulated by the 19 lncRNAs. We analysed the expression of 3 miRNAs (miR-21, miR-145, miR-148) and 5 genes (FGFR2, EGFR, KLF4, DNMT1, AGO4). Further, we carried out an integrated network analysis of lncRNA, miRNA and mRNA, to identify the existence of ceRNA networks in Indian gastric cancer.

## RESULTS

### Clinical characteristics

A total of 30 gastric tumor tissue samples with matched normal tissues adjacent to the tumor were used for the study. The mean age of the study group was 55.47 ± 13.1 and males were predominant (3:1). Almost 67 % (20/30) of the subjects were diagnosed with the tumor at pyloric antrum of the stomach and all of them were pathologically confirmed as adenocarcinoma. Of 30 gastric tumors, 47% (*n* = 14) of the patients were presented with higher tumors stage (>T2) and 83% are with nodal stage less than N2. Interestingly, 83% (*n* = 25) of the gastric tumors displayed undifferentiated cellular pathology. About 50% of the study population were positive for alcohol habit and 43% (13) had mixed risk habits (smoking, chewing and alcohol). Sixty percentage (*n* = 18) of the patients were non-vegetarians. The clinicopathological details of the tumor samples were given in Table 1.

**Table 1:** Clinical details of gastric cancer samples

Clinical characteristics		No. of patients (%)
**Age**	Mean ± SD	55.47 ± 13.1
	≤ 55 years	16 (48.6)
	> 55 years	14 (51.4)
**Gender**	Male	22 (74)
	Female	8 (26)
**Anatomical site**	Antrum	20 (67)
	Gastro-esophageal Junction	6 (20)
	Fundus body	4 (13)
**Sub-type**	Diffuse	20 (67)
	Intestinal	10 (33)
**Clinical stage**	≤ T2	16 (53)
	> T2	14 (47)
**Nodal metastasis**	≤ N1	25 (83)
	≥ N2	5 (17)
**Histological grade**	Well-differentiated	5 (17)
	Moderately differentiated	11 (36)
	Poorly differentiated	14 (47)
**Risk habit profile**	**Tobacco**	
	Non-user	13 (43)
	Smokers	12 (40)
	Chewers	5 (17)
	**Alcohol**	
	Non-Alcoholic	16 (53.3)
	< 10 Years	1 (3.3)
	>10 Years	13 (43.3)
	**Mixed habits**	
	Non-smoker/Non-alcoholic	12 (40)
	Smoking and chewing	5 (17)
	Smoking and alcoholic	10 (33)
	Smoking, chewing and alcoholic	13 (43)
	**Diet**	
	Non-vegetarian	18 (60)
	Vegetarian	12 (40)

### Identification of differentially expressed lncRNAs in gastric tumors

**Figure 1:**
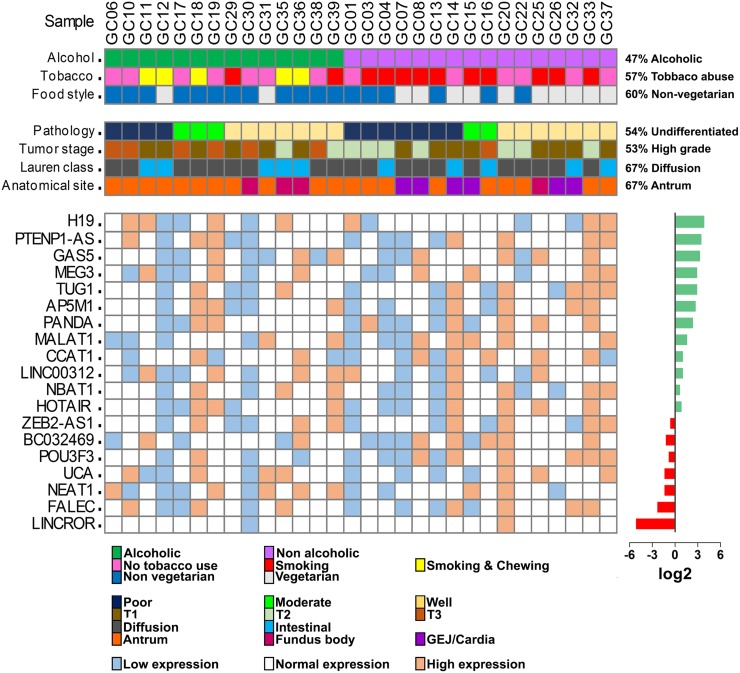
Oncoprint model of lncRNA expression in gastric tumor samples The top two panel shows the clinicopathological features of the 30 gastric cancer samples used in the study. The bottom heat map shows the expression levels of lncRNAs in each sample. The overall expression levels of the lncRNAs are presented in the right side of the expression heat map as log2 values.

To identify the deregulated expression of various lncRNAs in gastric cancer, we profiled the expression pattern of 19 lncRNAs, 7 oncogenic (H19, TUG1, CCAT1, HOTAIR, BC032469, NEAT1, FALEC), 2 tumor suppressive (PTENP1-AS, GAS5), 5 associated with cell proliferation (MEG3, AP5M1, LINC00312, NBAT1, POU3F3), 2 metastasis-associated (MALAT1, ZEB2-AS1), 1 apoptotic (PANDA), 1 associated with chemoresistance (UCA1) and 1 with reprogramming (LINCROR) shown to be dysregulated in various human cancers (Supplementary Table 1). In this study, we observed overexpression of 12 lncRNAs and 8 of them (H19, PTENP1-AS, GAS5, MEG3, TUG1, AP5M1, PANDA and MALAT1) were statistically significant (*P* < 0.05) and underexpression of 7 lncRNA of which 4 lncRNAs (LINCROR, FALEC, NEAT1 and UCA1) were statistically significant (Supplementary Table 2). The expression levels of the lncRNAs between samples were calculated to study the differential pattern of expression by classifying them into normal, low and high expression and compared with risk factors and clinicopathological features. The samples expressing between interquartile ranges (IQR) 25-75 were considered as normal expression, samples with less than 25 IQR as low expression and above 75 IQR as high expression (Figure 1).

**Figure 2:**
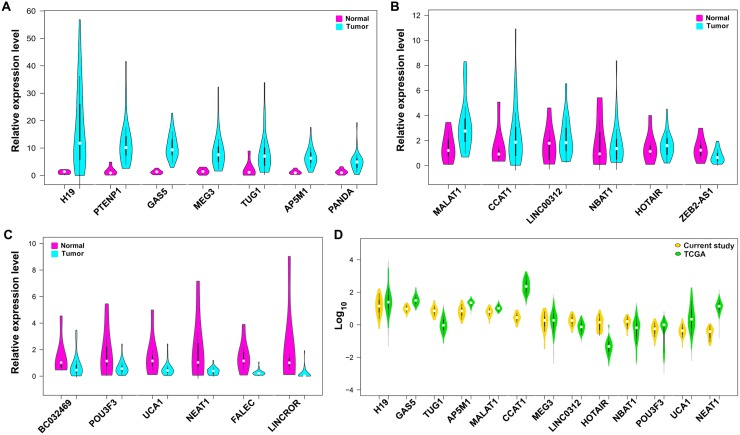
Expression level of lncRNAs in gastric tumors compared to adjacent normal tissues (**A**) Expression of lncRNAs that are classified as very highly expressed lncRNAs (>3 folds). (**B**) Expression of lncRNAs classified as highly expressed and (**C**) lncRNAs those are downregulated in tumors than normal tissues. (**D**) Comparative expression of lncRNAs from present study samples and TCGA gastric cancer datasets presented as log10 values.

Among the lncRNAs which were overexpressed, we classified them into three sub-categories namely very highly expressed (H19, PTENP1-AS, GAS5, MEG3, TUG1, AP5M1, PANDA), highly expressed (MALAT1, CCAT1, LINC00312, NBAT1, HOTAIR, ZEB2-AS1) and low level expressed (BC032489, POU3F3, UCA1, NEAT1, FALEC, LINCROR) (Figure 2A–2C). In addition, we analyzed the lncRNA levels of gastric cancer samples (*n* = 266) from the TCGA database. Consistent with our data, the lncRNAs H19, GAS5, TUG1, AP5M1, and MALAT1 were found to be overexpressed in TCGA database gastric tumors (Figure 2D). The RNA-Seq results from the public database TCGA-TANRIC database showed that MALAT1, H19, and TUG1 were among the top twenty overexpressed lncRNAs in gastric tumors [8]. The downregulated lncRNAs, LINCROR, POU3F3, HOTAIR, FALEC, NBAT1, and ZEB2-AS1 consistently matched with our study results while lncRNAs such as PTENP1-AS, AP5M1, PANDA, and NEAT1 are yet to be reported in the database (Supplementary Table 3).

We also investigated the association of lncRNA expression with the clinicopathological features and only Linc-POU3F3 was overexpressed in tumors from patients with non-vegetarian diet (*P* = 0.0430). We did not find any significant statistical association with other clinicopathological features with the lncRNAs expression (Supplementary Table 4). Similarly, when we checked the clinical association of the lncRNAs screened in the present study in TANRIC database, lncRNAs H19, MEG3 and MALAT1 showed significant association with tumor stage and HOTAIR had a significant association with patient survival (Supplementary Table 5).

### Differential expression of lncRNAs and mRNAs that share common MREs

Since the lncRNAs are differentially regulated and most of them are overexpressed several folds, it is possible that they can sponge miRNAs. Therefore, to investigate the miRNA-mediated regulatory interaction of lncRNAs and the downstream targets of the miRNAs, we collected the experimentally validated common miRNA targets from the public databases (LncBase Experimental v.2, TargetScan, MiRanda, and miRTarBase) (Supplementary Tables 6–11) and the lncRNAs that share MREs were obtained from Starbase, ceRDB, LncACTdb, miRSponge databases (Supplementary Tables 12–15). The data analysis identified that the three miRNAs (miR-21, miR-145, and miR-148a) and the five genes (EGFR, FGFR2, KLF4, DNMT1 and AGO4) are having an association with gastric malignancies. Therefore, the 3 miRNAs and 5 target genes were selected for expression profiling to elucidate their role in lncRNA-mediated ceRNA network in gastric cancer.

### Differentially expressed miRNAs strongly correlate with various clinical characteristics of gastric cancer

The expression levels of miR-21, miR-145 and miR-148a in gastric tumor samples were analyzed by miRNA assay using hydrolysis probe-based relative quantification method. Our results showed overexpression of miR-145, downregulation of miR-148a and differential expression of miR-21 (Supplementary Figure 1 and Supplementary Table 16A). Further, to explore the clinical association, the miRNA expression was compared with clinicopathological characteristics and a significant association of miR-145 expression with tumor stage (*P* = 0.0171), grade (*P* = 0.0245) and neoplastic score (*P* = 0.0455) was observed and miR-148a expression significantly correlated with non-alcoholics (*P* = 0.0470) and alcoholic patients (*P* = 0.0470) (Supplementary Table 16B).

### Expression profiling of gastric cancer associated genes harbouring common MRES

**Figure 3:**
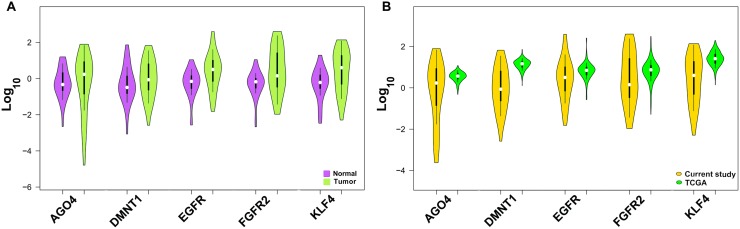
Expression level of gastric cancer-associated genes (**A**) Expression level of gastric cancer associated genes DNMT1, AGO4, KLF4, EGFR and FGFR2 between tumor and normal tissues. (**B**) Comparative expression of gastric cancer associated genes from present study samples and TCGA gastric cancer datasets.

We analyzed the expression level of gastric cancer-related genes EGFR, FGFR2, KLF4, DNMT1 and AGO4 identified through bioinformatics screening by relative quantification method and observed differential expression of EGFR, FGFR2, KLF4, DNMT1 and AGO4 in tumors. We found a significant overexpression of FGFR2 in gastric cancer samples with statistical significance (*P* = 0.0449) while other candidates did not show any statistical significance (Figure 3A and Supplementary Table 17A). The gene expression levels were analyzed for association with risk habits such as smoking and alcohol, and with the clinicopathological features; we did not observe any association (Supplementary Table 17B). Further, when the expression data of the present study was compared with the expression pattern of gastric cancer datasets from TCGA database, we found a similar expression pattern (Figure 3B).

### Identification of ceRNA networks

**Figure 4:**
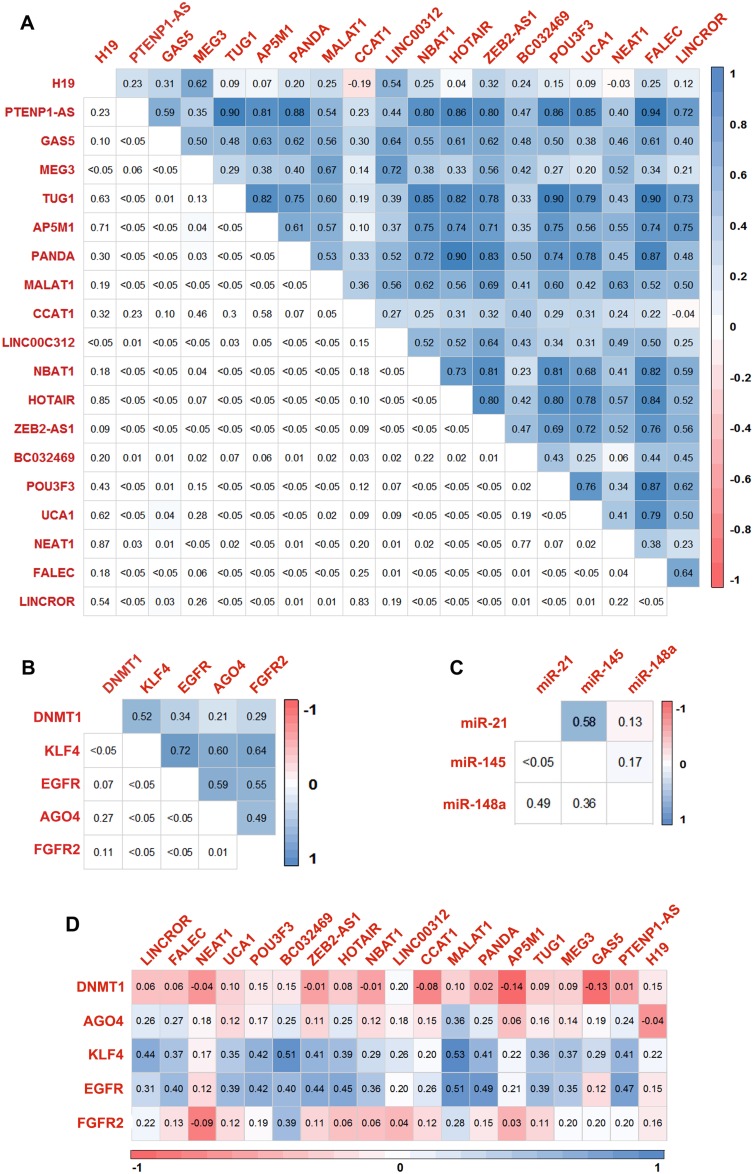
Correlation matrix of the genes screened for the expression Correlation matrix of differentially expressed lncRNAs (**A**), mRNA (**B**) and miRNA (**C**) in gastric cancer tissues based on spearman's linear correlation. (**D**) Correlation matrix showing the extent of correlation between the expression of lncRNAs and mRNAs in gastric tumors based on spearman's linear correlation.

**Figure 5:**
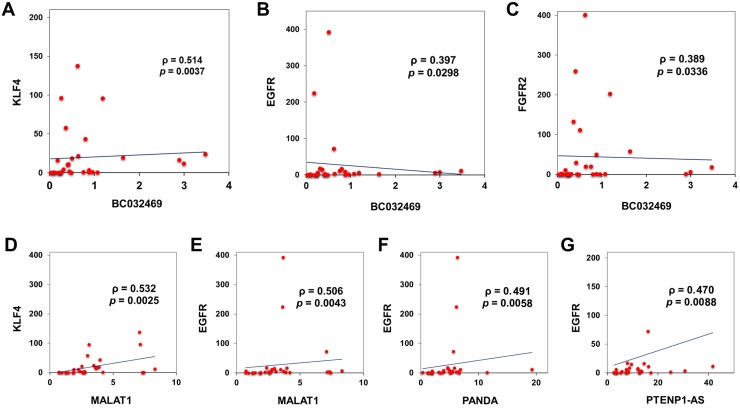
Linear regression analysis of lncRNA and mRNA Curve estimation linear regression analysis, coefficient analysis (slope) showing that BC032469 was positively associated with KLF4, EGFR and FGFR2 ((**A**–**C**) respectively) for the 30 gastric cancers included in the study. Similarly, linear regression analysis of KLF4 with MALAT1 (**D**) and EGFR with MALAT1 (**E**), PANDA (**F**) and PTENP1-AS (**G**), respectively showed a positive association.

**Figure 6:**
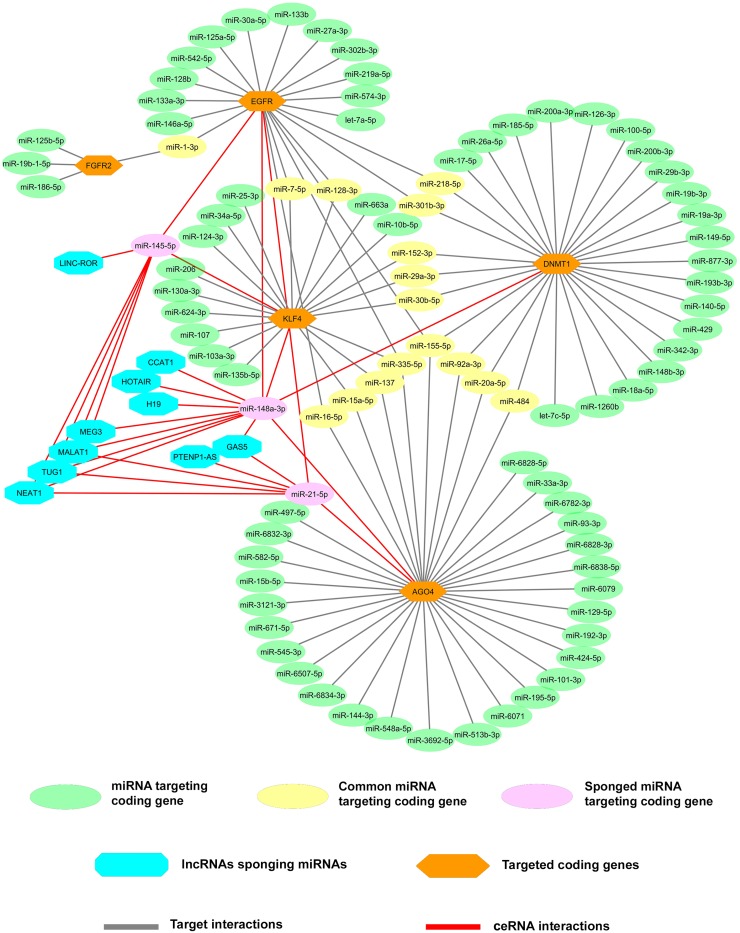
LncRNAs-miRNAs-mRNAs co-expression network for identification of novel ceRNA network A prediction of the ceRNA hypothesis in gastric cancer by integrated analysis of the mRNAs and lncRNAs containing the same MREs which compete for a common pool of miRNAs using online prediction tools and validated reports. In the ceRNA network, the lncRNAs (blue node) can sponge the miRNAs (pink node) that target the gastric cancer driver or associated genes (orange node) and enables the genes to skip miRNA-mediated post-translation gene regulation. The miRNAs those not sponged by lncRNAs that were targeting individual genes were mentioned in the green node and miRNAs targeting two or more genes were mentioned in the yellow node.

To ascertain the molecular interactions between lncRNA, mRNA, and their common miRNA targets, correlation analysis was performed to check the association of gene expressions. Most of the lncRNAs are found to have a positive correlation with significant *P*-value (Figure 4A). Similarly, the mRNAs and miRNAs also showed a significant correlation (Figure 4B and 4C). We also performed Spearman correlation test between lncRNAs and mRNAs and observed that BC032469, MALAT1, PTENP1-AS, and PANDA had a significant correlation with gastric cancer-associated genes (Figures 4D, 5 and Supplementary Table 18). Further, an integrated analysis was performed by computational network analysis and the network was visualized using CYTOSCAPE. The *in-silico* network analyses predicted that the deregulated lncRNAs H19, PTENP1-AS, TUG1, GAS5, MALAT1, and LINCROR could sponge the miR-148a, miR-145 and miR-21 and can act as ceRNA modulating the miRNA-mediated post-transcriptional regulation of EGFR, KLF4, DNMT1 and AGO4 (Figure 6). In the ceRNA network, orange colored nodes represent gastric cancer associated mRNAs; pink nodes represent miRNAs involved in ceRNA network (interactions represented by red lines); blue nodes represent lncRNAs; yellow nodes represent miRNAs targeting more than one target mRNA and green colored edges indicate other miRNA-target interactions.

### Sponging of candidate miRNAs by overexpressed lncRNAs

Comparative analysis of the KLF4, TUG1, and miR-145 suggested a significant relationship between these three different RNAs (Supplementary Figure 2A). In addition, we found the downregulation of LINCROR when its target miR-145 abundantly expressed. Further, the expression pattern indicated that MALAT1 synchronize the expression level of KLF4 and miR-145 and MALAT1 had a strong impact than TUG1 in regulating KLF4 through sponging miR-145. H19 is a well-known oncogenic lncRNA that is involved in the reduction of p53 activity inducing proliferation in gastric cancer [9]. In the present study, we observed a higher level of H19 which sponged out the low level expressed miR-148a leading to the overexpression of its target mRNA DNMT1 (Supplementary Figure 2B). Similarly, we also observed the KLF4 overexpression due to the H19 mediated sponging of miR-148a (Supplementary Figure 2C). On the other hand, it is reported that PTENP1-AS pseudogene interaction with miR-21 leading to EGFR overexpression and AGO4 is another target of miR-21 [10]. In this study, we observed upregulation of PTENP1-AS increased the EGFR and AGO4 level in tumors. In addition, we also observed that lncRNA GAS5 competes with AGO4 for miR-21. Compared to PTENP1-AS, the GAS5 predominantly overexpressed in this study samples and the expression level was comparatively higher than competing mRNA AGO4.

## DISCUSSION

Gastric cancer, a severe life-threatening cancer in global population and remains a major health burden in Asian countries including India. Unlike another type of cancers, it is often identified at an advanced stage which delimits the clinical management. Early molecular events of gastric tumorigenesis are characterized by complex genomic interactions. Recently, non-coding RNAs (ncRNAs) are established as key players in different cancers including gastric malignancies [11–13]. The lncRNAs have been shown to regulate the miRNA activity, the post-transcriptional regulation affecting its target mRNA turnover and equilibrium in a normal cell [14]. This study is an attempt to delineate the ceRNA mediated molecular mechanism operating in the gastric tumorigenesis at the transcriptional level by analyzing the regulatory interaction between non-coding and protein-coding RNAs.

To determine the deregulated lncRNAs in gastric cancers, we profiled the expression pattern of 19 human cancer-associated lncRNAs and found a statistically significant overexpression of 8 lncRNAs (H19, PTENP1-AS, GAS5, MEG3, TUG1, AP5M1, PANDA and MALAT1) and significant downregulation of 4 lncRNAs, LINCROR, FALEC, NEAT1 and UCA1. Further, we observed the association of Linc-POU3F3 overexpression in gastric tumors from patients with non-vegetarian diet habit (*P* = 0.043). Consistent with our results, we also found overexpression of lncRNAs such as H19, GAS5, TUG1, AP5M1 and MALAT1 and downregulation of LINCROR, POU3F3, HOTAIR, FALEC, NBAT1, and ZEB2-AS1 lncRNAs in TCGA gastric tumor datasets. H19 has been characterized as an oncogenic lncRNA and shown to be overexpressed in four gastric cancer cell lines (AGS1, MGC803, SGC7901, and MKN45). Functional validation of high level of H19 has been found to inhibit p53 activity that favored gastric cancer progression [9]. Decreased levels of MEG3 have been often linked with oncogenesis and progression of lung, bladder, kidney, and hepatocellular carcinoma. Though MEG3 downregulation was reported in some cases of gastric cancers, we observed overexpression of MEG3 in gastric tumors in this study [15]. Similarly, overexpression of UCA1 induced by SP1 was reported to promote cell proliferation via recruiting EZH2 and activating AKT pathway in gastric cancer [16]. To the contrary, we report significant downregulation of UCA1 in the present study population. The exact molecular mechanism behind the differential expression of MEG3 and UCA1 could be due to ethnic difference and the etiological factors specific to Indian gastric cancer patients.

Consistent with the present data, TUG1, PANDA, and MALAT1 was shown to be overexpressed in several studies. TUG1 has recently been shown to express at a significantly high level and predicts the overall survival of gastric cancer patients [17]. PANDA upregulation has been shown to associate with invasiveness and prognosis of gastric cancer. Further, PANDA overexpression was shown to be associated with poor survival and considered as an independent predictor of overall survival and clinical outcome of patients [18]. We observed ~50% of our tumor samples with non-metastatic tumors had upregulated MALAT1. Aberrant MALAT1 levels have been observed to alter the stability of mRNA splicing proteins SF2/ASF that leads to their dysfunction, an important event needed for the cellular proliferation of gastric cancer [19]. MALAT1 expression has been observed to be correlated with peritoneal dispersion in gastric cancer suggesting that tumors with MALAT1 might be on the verge of metastatic progression [20]. Apart from oncogenic lncRNA signatures, our study samples also exhibited overexpression of some tumor suppressor lncRNAs (PTENP1-AS and GAS5). PTENP1, a pseudogene regulates cellular PTEN levels and plays a vital role in growth suppression. Circulating PTENP1-AS has been identified to serve as a serum marker to differentiate gastric cancer patients from healthy controls [21]. GAS5 has been reported to be upregulated in cells under growth inhibition caused by starvation and it acts as a tumor suppressor by downregulating oncogenic CDK6 (cyclin-dependent kinase-6) in pancreatic, bladder and gastric cancer cells and involved in G1 phase cell cycle arrest by interacting with transcriptional activator YBX1 in stomach cancer [22, 23]. Both the upregulated and downregulated lncRNAs which we observed in the current study may play a crucial role in gastric cancer progression.

LncRNA based destabilization of miRNA is recently implicated. Hence, we hypothesized that post-transcriptional interaction between mRNA-miRNA could be interrupted by some tumor-associated lncRNA in gastric cancer. We screened for the candidate miRNAs which are having common MREs with gastric cancer-associated genes and interaction with lncRNAs. We found 3 miRNAs, miR-21, miR-145, and miR-148a involved in the post-transcriptional regulation of the candidate genes, and shared MREs with the differentially expressed lncRNAs analyzed in this study. Expression analysis of these miRNAs in our study showed overexpression of miR-145, downregulation of 148a and differential expression of miR-21. Moreover, we found a significant statistical association of tumor stage (*P* = 0.0171), grade (*P* = 0.0245) and neoplastic score (*P* = 0.0455) with elevated expression of miR-145 suggesting the significance of miR-145 in gastric cancer tumor progression. We also observed a significant correlation of miR-148a upregulation in the patient with alcohol history (*P* = 0.0470), suggesting its association with alcohol-induced gastric tumorigenesis.

In addition to the lncRNAs, we also profiled the expression pattern of identified candidate gastric cancer-associated genes EGFR, FGFR2, KLF4, DNMT1 and AGO4 shortlisted from the analysis of genes by relative quantification. Although we found a differential expression of EGFR, FGFR2, KLF4, DNMT1 and AGO4 in gastric cancer samples, only FGFR2 overexpression was statistically significant (*P* = 0.0449). Consistently, we found similar results when we analyzed the data derived from TCGA gastric cancer datasets. The mRNA candidates analyzed in the study were selected based on their expression signature that has been well characterized with clinical features of gastric cancer [9, 10, 24]. Also, each of the mRNA has been linked with gastric cancer prognosis by exhibiting different molecular role in gastric tumorigenesis.

Further, we identified the interaction between the overexpressed lncRNAs and miRNAs that affected the expression level of coding genes. Interestingly, our study predicted that the deregulated lncRNAs such as H19, PTENP1-AS, TUG1, GAS5, MALAT1 and LINCROR could act as competing endogenous RNA by sponging the miR-148a, miR-145 and miR-21 resulting in the upregulation of EGFR, KLF4, DNMT1 and AGO4 mRNA levels. This study identifies the gastric cancer-associated genes such as EGFR, KLF4, DNMT1 and AGO4 as candidates of ceRNA network in gastric cancer.

In addition, we identified several ceRNA networks associated with miR-148a, miR-145, and miR-21. In addition, we observed overexpression of H19 and DNMT1 when their targeting miR-148a expressed at lower levels in tumors. Similarly, we also observed the KLF4 overexpression that might be due to the overexpressed H19 mediated sponging of miR-148a. These results suggest that elevated level of H19 could sponge miR-148a which in turn allows both DNMT1 and KLF4 free from miRNA-mediated regulation leading to tumorigenesis [24]. Similarly, other overexpressed lncRNAs namely TUG1 and MALAT1 were also found to act as ceRNA in bladder cancer and cervical cancer [25, 26]. Competing endogenous activity of H19 identified in this study suggests that overexpression of H19 and miR-148a downregulation could be a signature for gastric cancer.

In this study, a significant relationship was observed between the KLF4, TUG1 and miR-145. TUG1 and MALAT1 overexpression directly correlated with KLF4 and EGFR expression. In addition, we observed the expression level LINCROR directly correlated with the overexpression of the miR-145. This relationship has been already reported in colon cancer and shown to be involved in overall survival [27]. Our results suggest that reduced LINCROR/high miR-145 might be associated with better prognosis. LINCROR is an active member associated with reprogramming and stemness-related transcription factors. Downregulation of LINCROR by miR-145 might impair the cancer stemness by deregulation of another transcription factor KLF4 [28, 29].

Overexpression of tumor suppressor lncRNAs PTENP1-AS and GAS5 might, in turn, reduce the oncogenic role of miR-21 [30, 31]. We observed upregulation of PTENP1-AS that could increase the EGFR and AGO4 expression in tumors and also noticed that GAS5 could compete with AGO4 and regulate the miR-21. Compared to PTENP1-AS, the GAS5 expression was predominantly upregulated in the gastric tumor samples and the expression level was comparatively higher than competing mRNA AGO4. Collectively, these observations suggest that overexpression of PTENP1-AS and GAS5 in gastric cancer could be abrogating oncogenic activity of miR-21 and confirms that lncRNAs could act as tumor suppressive ceRNAs.

In summary, our study results showed differentially regulated lncRNAs and gastric cancer-associated genes such as EGFR, KLF4, DNMT1 and AGO4 are candidates of ceRNA network in gastric cancer. All the 27 genes analyzed were selected individually based on the earlier reports that demonstrated the association of our candidate genes in a different type of cancers including gastric cancer (Table 2). Further, the correlation analysis between the lncRNAs and target mRNAs showed the involvement of novel ceRNA networks in gastric cancer. Moreover, we prioritized our study to figure out the overexpressed lncRNA candidate may have any role in sponging activity by absorbing some common miRNA targets. With the assumption of single miRNA can target many candidate genes, we performed ceRNA network analysis and analyzed three miRNAs miR-21, miR-145 and miR-148a whose expression signature has been already reported in gastric cancer. Our analysis revealed that the 3 miRNAs are central candidates that are coordinated by sponging activity of lncRNAs analyzed in this study. The ceRNA network identified in this study could be used to understand the molecular mechanism of gastric tumorigenesis. It is evident that negative and positive correlation is always possible in ceRNA mechanism where we paid attention towards positively correlated lncRNAs as most of our candidates are overexpressed. It is acknowledged that preliminary high-throughput screening methods like microarray or RNAseq could be convincing for the precise selection of the candidates. On the other hand, covering entire normal transcriptome and cancer transcriptome in respective gastric tissue biopsies needs more attention even it was performed with few selected representative samples. Considering our limitation of performing high-throughput techniques for discovery studies, we aimed to focus on few candidates already well characterized in gastric cancer. Due to the limitations of experimental methods in understanding the relationship between RNA networks and diseases, several computational methods were developed [32]. Cross-validation of heterogeneous networks across cancers using data available in public data sources using machine learning and advanced computational methods may resolve these limitations [33]. Further, expression profiling with a larger number of tumor samples and functional characterization is warranted to delineate the differential role of the lncRNAs and the ceRNA networks in gastric cancer. The functionally validated lncRNAs involved in ceRNA network could serve as prognostic indicators and/or therapeutic targets for gastric cancer.

**Table 2:** List of genes analysed in this study and their role in cancers

Candidate gene		Type of Cancer/Function	Reference
LncRNA	H19	Gastric- Oncogenic	Yang F *et al*. (2012)
	HOTAIR	Gastric- Oncogenic	Endo H *et al*. (2013)
	CCAT1	Gastric- Oncogenic	Yang F *et al*. (2013)
	GAS5	Gastric-Tumor suppressor	Sun M *et al*. (2014)
	MALAT1	Gastric-Oncogenic	Okugawa Y *et al*. (2014)
	MEG3	Gastric-Tumor suppressor	Peng W *et al*. (2015)
	UCA1	Gastric-Oncogenic	Zheng Q *et al*. (2015)
	POU3F3	Gastric-Oncogenic	Xiong G *et al*. (2015)
	TUG1	Gastric-Oncogenic	Zhang E *et al*. (2016)
	BC032469	Gastric-Oncogenic	Lu M *et al*. (2016)
	PTENP1	Gastric-Tumor suppressor	Guo X *et al*. (2016)
	PANDA	Gastric-Oncogenic	Ma P *et al*. (2016)
	NEAT1	Gastric-Oncogenic	Fu J *et al*. (2016)
	LINC00312	Nasopharyngeal-Tumor suppressor	Zhang W *et al*. (2013)
	AP5M1	Nasopharyngeal-Tumor suppressor	Wang Q *et al*. (2014)
	NBAT1	Neuroblastoma-Tumor suppressor	Pandey G *et al*. (2014)
	ZEB2-AS1	Hepatocellular-Oncogenic	Li T *et al*. (2016)
	FALEC	Prostate-Oncogenic	Zhao R *et al*. (2017)
	LINCROR	Oral-Oncogenic	Arunkumar G *et al*. (2017)
miRNA	miR-21	Gastric-OncoMir	Zhang Z *et al*. (2008)
	miR-145	Gastric-Tumor suppressor	Takagi T *et al*. (2009)
	miR-148a	Gastric-Tumor suppressor	Zhu A *et al*. (2012)
mRNA	DNMT1	Methylation	Etoh T *et al*. (2004)
	KLF4	Growth arrest	Wei D *et al*. (2005)
	EGFR	Communication	Galizia G *et al*. (2007)
	FGFR2	Communication	Kunii K *et al*. (2008)
	AGO4	miRNA processing	Wang Y *et al*. (2012)

## METHODS

### Tumor tissue samples

Tumors with adjacent normal tissues (*n* = 30) were collected from patients undergoing curative surgical gastrectomy at Department of Surgical Gastroenterology, Rajiv Gandhi Government General Hospital, Madras Medical College, Chennai, Tamil Nadu, India. Institutional Ethics Committee (IEC) approval for the study was obtained (IEC No.14597/ME5/Ethics Dean/MMC/2010) and the ethics guideline was strictly followed in this study. All the patients were explained about the purpose of the research study and a written informed consent was obtained before the sample collection. The demographic and clinicopathological details were obtained from the hospital records. Tissue specimens were collected in 5 ml screw cap vials (Genaxy, India) containing 3 mL of RNA stabilization reagent RNA*later* (Ambion, USA) and transported on gel ice pack to the laboratory.

### RNA isolation and cDNA synthesis

Tissues soaked in RNA*later* were washed twice with ice-cold PBS to remove the residual stabilizing solution. Tumor and normal tissue (~100 mg) were homogenized in bead-based homogenizer MicroSmash™ MS-100 (Tomy Digital Biology, Japan) in a pulsating manner with 3500 rpm for 30 seconds (3–4 times) with intermediate incubations on ice for 5 minutes. RNA was isolated using RNeasy kit (Qiagen, Germany) following the manufacturer's protocols and eluted in 30 μL nuclease-free water and stored at −80° C. The quantity and quality of isolated RNA samples were evaluated using Nanodrop 2000 UV-Vis Spectrophotometer (Thermo Scientific, Germany) and by resolved in 1% agarose gel containing 0.5 μg/mL ethidium bromide in Mupid gel electrophoresis system (TaKaRa, Japan), respectively.

cDNA was synthesized by using three different primers (i) Universal Oligo (dT) primer for expression profiling of lncRNA (ii) random hexamer primer for mRNA expression analysis and (iii) miRNA-specific stem-loop RT primer for miRNA expression (Supplementary Tables 19 and S20). Two-step cDNA conversion reaction was performed using Invitrogen Reverse Transcription Kit (Invitrogen, USA) as per manufacturer's instructions using total RNA. The RNA was mixed with respective primer and pre-incubated at 65° C for 20 minutes to denature the RNA secondary structures and placed on ice. The RT-master mix containing 5X RT Buffer, 0.5 mM dNTP mix, 1 mM DDT and 200 U of SuperScript^®^ III (Invitrogen, USA) was added and made to a final volume of 20 μl with RNase-free water. For the first strand synthesis, the samples were incubated at 50° C for 90 min, 70° C for 15 min, and finally, at 4° C. The cDNAs were diluted to 1:50 with nuclease-free water and stored at −20° C.

### Real-time quantitative PCR

Relative Quantification (RQ) was performed using the hydrolysis probed based custom assays (ABI, USA). LncRNA forward primers specific to the last exon/3’end of the lncRNAs and a universal reverse primer were used for the lncRNAs. MiRNA-specific forward primer and a universal reverse primer were used to profile the mature miRNAs. RT-qPCR was carried out in 384 well optical plates (in triplicates) in 10 μL reaction with Roche Fast Start Universal Master Mix (Roche Applied Science, Germany) using specific forward primer, universal reverse primer and universal FAM labelled MGB probe (5ʹ-CAGAGCCACCTGGGCAATTTT-3ʹ) for lncRNAs and miRNAs. Predesigned assays for GAPDH, DNMT1, AGO4, KLF4, EGFR and FGFR2 gene expression analysis were procured from Life Technologies Inc., USA. All the gene expression assays were carried out by following supplier's protocol using 7900 HT Real-Time PCR System (ABI, USA). A negative control without cDNA was also included for all the assays. GAPDH and RNU44 were used as endogenous references for lncRNAs/mRNAs and for miRNAs respectively. The expression level was calculated using 2^−ΔΔCt^ method [34].

### Development of ceRNA network

ceRNA network was constructed with the following four steps: (1) lncRNAs which are up or downregulated and exhibited >2.0 fold change were selected and their functionally proven target miRNAs were retrieved from a public database using DIANA Tools and LncBase Experimental v.2 (http://www.microrna.gr/LncBase/), (2) mRNAs which are deregulated with a fold change >2.0 were taken and their experimentally proven target miRNAs were collected from three different public databases like Target Scan (Release 7.0) (http://www.targetscan.org), DIANA-TarBase (v7.0) (http://diana.imis.athena-innovation.gr/), miRTarBase (Release 6.0) (http://mirtarbase.mbc.nctu.edu.tw/), (3) The common miRNA targets between these lncRNA-mRNA were filtered manually, (4) The ceRNA interaction network was constructed using Cytoscape version 3.4.1 (Institute for system biology, Seattle, USA). The interaction network was designed in three phases, initially, lncRNA-miRNA interactions were networked and subsequently mRNA-miRNA interactions were created. Finally, the two separate networks (lncRNA-miRNA & mRNA-miRNA) were merged together to visualize the overall interaction between the candidate mRNAs, targeted by miRNAs and lncRNA. The edges were assigned for mRNAs & lncRNAs and the nodes were designated for the common miRNAs between the mRNA & lncRNAs that might serve as ceRNA.

### Statistical analysis

Statistical analyses were performed using GraphPad Prism 6 (GraphPad Software Inc., La Jolla, CA, USA) for validating expression level of lncRNA, mRNA, and miRNA. Relative expression of tumors associated 19 selected lncRNAs was statistically compared with adjacent normal tissues. The expression data were log2 transformed and tested for normality with the Kolmogorov-Smirnov test. The difference between the variance was analyzed using Student's *t*-test. All the tests were two-tailed and a *p* < 0.05 was considered significant.

## SUPPLEMENTARY MATERIALS FIGURES AND TABLES


