# Supplementary material for: Comprehensive analysis of aberrantly expressed lncRNAs and construction of ceRNA network in gastric cancer
Source: Oncotarget. 2018 Apr 6;9(26):18386–99. doi: 10.18632/oncotarget.24841 (PMC5915079; doi:10.18632/oncotarget.24841)
Supplement: Supplementary file 1 [file oncotarget-09-18386-s001.pdf]

Comprehensive analysis of aberrantly expressed lncRNAs and construction of ceRNA network in gastric cancer

SUPPLEMENTARYARY MATERIALS

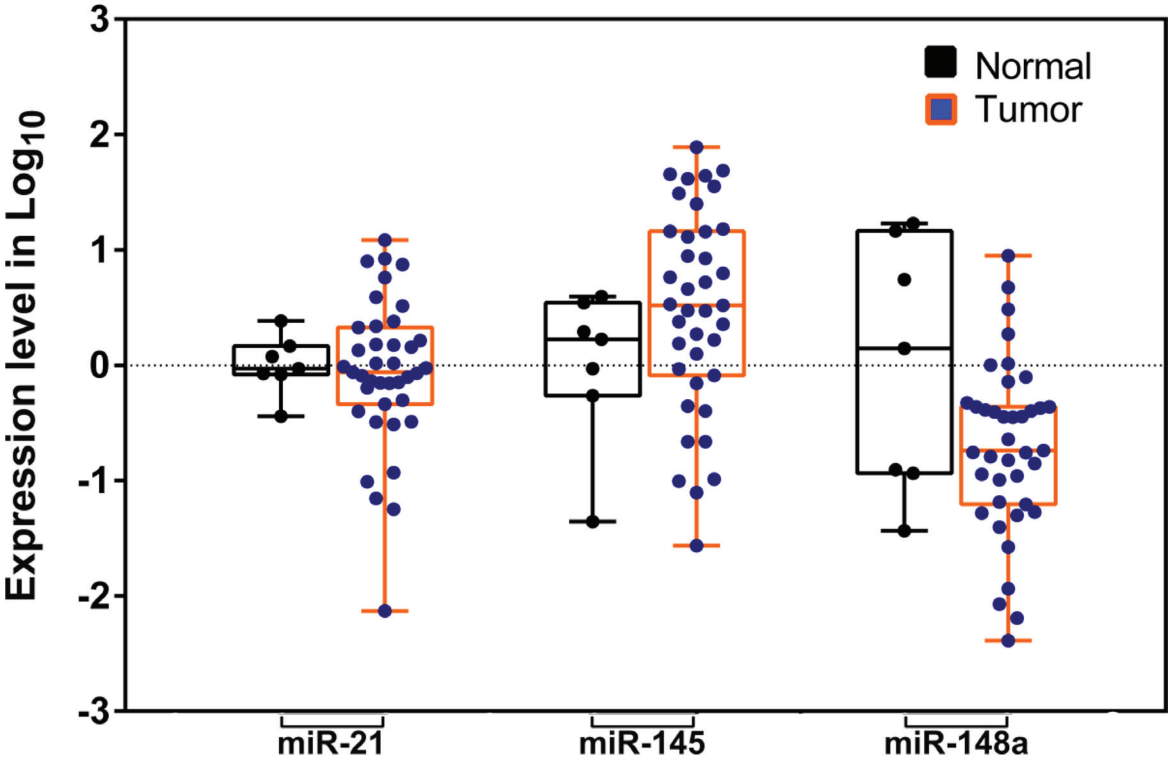

Supplementary Figure 1: Expression levels of microRNAs those potentially involved in ceRNA network. Expression levels of miR-21, miR-145 and miR-148a in 30 gastric tumors and adjacent normal tissues. miR-21 and miR-145 were overexpressed whereas miR-148a was downregulated in gastric tumor compared to normal tissue.

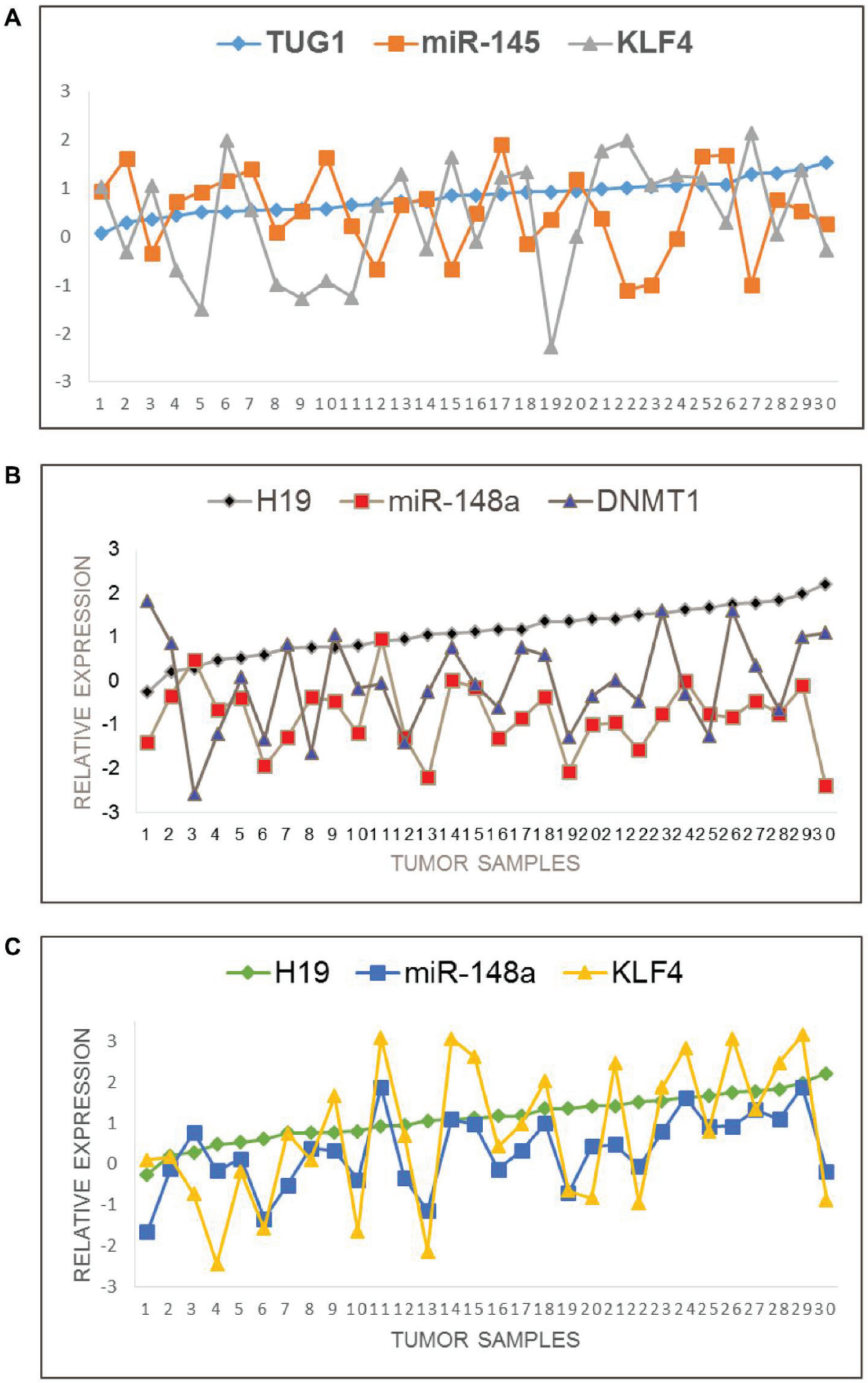

Supplementary Figure 2: Comparative expression of lncRNA, miRNA and mRNAs involved in competing endogenous network in gastric cancer. ceRNA network modulated by lncRNA TUG1 through sponging miR-145 which targets KLF4 mRNA (**A**). Similarly, ceRNA network exhibited by lncRNA H19 by absorbing miR-145 which targets DMNT1 and KLF4 in gastric tumors (**B** and **C**, respectively).

Supplementary Table 1: List of lncRNAs reported in various human cancers

Candidate	Type of Cancer	Reference	PMID
H19	Gastric	Yang F (2012)	22776265
HOTAIR	Gastric	Endo, H (2013)	24130837
CCAT1	Gastric	Yang F (2013)	23143645
GAS5	Gastric	Sun M (2014)	24884417
MALAT1	Gastric	Okugawa Y (2014)	25280565
MEG3	Gastric	Peng W (2015)	26253106
UCA1	Gastric	Zheng Q (2015)	25903045
POU3F3	Gastric	Xiong G (2015)	26807174
TUG1	Gastric	Zhang E (2016)	26913601
BC032469	Gastric	Lu M (2016)	26549025
PTENP1-AS	Gastric	Guo X (2016)	25968876
PANDA	Gastric	Ma P (2016)	26898439
NEAT1	Gastric	Fu J (2016)	27095450
LINC00312	Nasopharyngeal	Zhang W (2013)	23529758
AP5M1	Nasopharyngeal	Wang Q (2014)	24379026
NBAT1	Neuroblastoma	Pandey G (2014)	25517750
ZEB2-AS1	Hepatocellular	Li T (2016)	27748842
FALEC	Prostate	Zhao R (2017)	28585762
LINROR	Oral	Arunkumar G (2017)	28443494

Supplementary Table 2: *P*-values of lncRNAs between tumor and normal

LncRNA	*P* value	*P* value summary
H19	<0.0001	****
PTENP1-AS	<0.0001	****
GAS5	<0.0001	****
MEG3	<0.0001	****
AP5M1	<0.0001	****
PANDA	0.0002	***
TUG1	0.0009	***
MALAT1	0.0116	*
CCAT1	0.2712	ns
LINC00312	0.3716	ns
NBAT1	0.9103	ns
HOTAIR	0.4699	ns
ZEB2-AS1	0.125	ns
BC032469	0.0578	ns
POU3F3	0.0578	ns
UCA1	0.0185	*
NEAT1	0.0208	*
FALEC	0.0013	**
LINCROR	<0.0001	****

$^{*}P$ value with statistical significance. $^{*}P < 0.05$, $^{**}P < 0.01$, $^{***}P < 0.001$ and $^{****}P < 0.0001$ vs. normal tissues.

Supplementary Table 3: LncRNA expression in gastric cancer samples from TANRIC database

**Top 20 Overexpressed lncRNAs in gastric cancer**		
**ENSEMBL ID**	**lncRNA**	**Mean log2**
ENSG00000269888.1	lnc-OTOL1-5	7.111
ENSG00000258486.2	lnc-LRR1-1:1	6.462
ENSG00000251562.3	MALAT-1[$]	5.943
ENSG00000262477.1	lnc-MEP1B-2:1	5.369
ENSG00000177410.8	ZFAS1	5.311
ENSG00000260032.1	NORAD:3	4.697
ENSG00000248223.1	lnc-BASP1-1:1	4.457
ENSG00000269972.1	lnc-MORC2-1:1	4.120
ENSG00000245910.4	SNHG6:2	3.998
ENSG00000175061.13	LRRC75A-AS1:75	3.982
ENSG00000269987.1	lnc-MORC2-2:1	3.566
ENSG00000226950.2	DANCR:8	3.555
ENSG00000225733.1	FGD5-AS1:4	3.507
ENSG00000269893.2	SNHG8:2	3.395
ENSG00000232388.2	LINC00493:4	3.333
ENSG00000240567.1	lnc-OTOL1-1:1	3.295
ENSG00000130600.11	H19:14[$]	3.287
ENSG00000253352.4	TUG1:19[$]	3.275
ENSG00000203875.6	SNHG5:4	3.223
ENSG00000269900.2	lnc-C9orf100-1:2	3.134
**Remaining candidates of the study**		
ENSG00000234741	GAS5	2.712
ENSG00000247844.1	CCAT1	–0.697
ENSG00000214049.6	UCA1	–0.793
ENSG00000237697	LINC312	–1.616
ENSG00000214548.10	MEG3	–1.643
ENSG00000228630.1	HOTAIR	–4.440
ENSG00000228126.1	FALEC	–5.018
ENSG00000249201.2	BC032469	–6.282[b]
ENSG00000260455.1	NBAT1	–6.309
ENSG00000238057.8	ZEB2-AS1	–7.720
ENSG00000258609.1	LINCROR	–12.624
ENSG00000228528.1	POU3F3	–15.575
ENSG00000281128.1	PTENP1-AS	N/A
ENSG00000053770	AP5M1	N/A
ENSG00000281450.1	PANDA	N/A
ENSG00000245532.5	NEAT1	N/A

[$]lncRNAs screened in present study listed in top 20 overexpressed lncRNAs in gastric cancer obtained using TANRIC database.

Supplementary Table 4: Association of lncRNAs expression with tumor clinicopathological features

LncRNA	Diffuse Vs. Intestinal	< Stage II Vs. > Stage II	<T2 Vs. >T2	<N2 Vs. >N2	Tobacco user Vs. Non-user	Alcoholic Vs. Non-alcoholic	Veg Vs. Non-Veg
H19	0.9096	0.8738	0.8450	0.3739	0.8553	0.6602	0.9337
PTENP1-AS	0.5861	0.6022	0.9704	0.6240	0.7662	0.9736	0.1991
GAS5	0.3410	0.2889	0.7507	0.9489	0.5867	0.7790	0.5980
MEG3	0.9999	0.9090	0.6309	0.3083	0.7977	0.9736	0.0863
TUG1	0.7424	0.9736	0.6872	0.7153	0.3822	0.4659	0.0946
AP5M1	0.9779	0.2798	0.4057	0.9489	0.7662	0.7790	0.0786
PANDA	0.7101	0.8780	0.5182	0.7625	0.7662	0.9736	0.4368
MALAT1	0.9437	0.9381	0.6872	0.8107	0.2983	0.4173	0.0587
CCAT1	0.9999	0.2615	0.1446	0.6690	0.5047	0.9704	0.1991
LINC312	0.9437	0.6899	0.8450	0.9989	0.5867	0.9704	0.4619
NBAT1	0.6643	0.9999	0.4413	0.5693	0.7977	0.8769	0.4125
HOTAIR	0.8418	0.8133	0.6283	0.9989	0.4052	0.6872	0.1578
ZEB2-AS1	0.9437	0.8419	0.6283	0.8495	0.8234	0.7202	0.2475
BC032469	0.0672	0.7819	0.6309	0.7625	0.3822	0.6872	0.0786
POU3F3	0.8083	0.8627	0.4058	0.9585	0.1823	0.4913	0.0430[#]
UCA1	0.7424	0.8627	0.1001	0.4578	0.7917	0.9736	0.3890
NEAT1	0.9617	0.9090	0.7790	0.8107	0.6989	0.8769	0.4125
FALEC	0.5861	0.8769	0.6872	0.8990	0.7351	0.9381	0.3663
LINCROR	0.4209	0.6602	0.8450	0.5801	0.8294	0.7790	0.5143

[#] *P* value with statistical significance.

Supplementary Table 5: Clinical association of LncRNAs from TANRIC database

lncRNAs	Lauren	Tumor stage	Survival		Correlation		
			Cox-*P* value	log Rank *P*-Value	miRNA	r^2	*P*-value
HOTAIR	0.5976	0.0996	0.0380#	0.1350	miR-196b	0.56	0.0000
GAS5	0.0000	0.1861	0.4187	0.9685	miR-17	0.52	0.0000
LINC00312	0.3763	0.2725	0.5632	0.1730	miR-215	0.47	0.0000
CCAT1	0.0009#	0.6535	0.8358	0.5842	miR-584	0.42	0.0001#
ZEB2-AS1	0.0035#	0.1824	0.8985	0.3090	miR-200b	–0.48	0.0000
TUG1	0.0003#	0.7647	0.4229	0.9638	miR-1538	–0.46	0.0000
H19	0.7261	0.0021#	0.8494	0.6681	miR-148a	–0.41	0.0002#
MEG3	0.0002#	0.0249#	0.8222	0.8849	miR-148a	–0.41	0.0002#
LINCROR	0.1062	0.4012	0.8090	0.8347	miR-1185-1	–0.40	0.0002#
MALAT1	0.1254	0.0270#	0.9699	0.2557	n/a	n/a	n/a
NEAT1	0.0595	0.1186	0.7438	0.5063	n/a	n/a	n/a
UCA1	0.0000	0.2404	0.6247	0.8134	n/a	n/a	n/a

#*P* value with statistical significance.

Supplementary Table 6: LncRNAs targeted by miRNA by LncBase experimental v.2

NCBI ID	LncRNA Name	miRNA name	Method	Score
NR_002196.1	H19	miR-148a-3p	IP	0.657
NR_103745.2	PTENP1-AS	miR-21-5p	RS	–
NR_002578.2	GAS5	miR-21-5p	qPCR	0.382
		miR-148a-3p	IP	0.506
NR_002766.2	MEG3	miR-145-5p	IP	0.771
		miR-148a-3p	IP	0.597
NR_110492.1	TUG1	miR-21-5p	IP	0.68
		miR-145-5p	IP	0.959
		miR-148a-3p	IP	0.676
NR_002819.2	MALAT-1	miR-21-5p	IP	0.611
		miR-145-5p	IP	0.519
		miR-148a-3p	IP	0.551
NR_028272.1	NEAT1	miR-21-5p	IP	0.406
		miR-145-5p	IP	0.649
		miR-148a-3p	IP	0.796
NR_048536.1	LINC-ROR	miR-145-5p	qPCR	0.585

Supplement Table 7: LncRNAs targeted by miRNA by MiRanda tool

<table>
<tr><th>microRNA interaction with LncRNA</th><th>mirSVR score</th></tr>
<tr><td>3′ ucCCUAAGGACCCUUUUGACCUg 5′ hsa-miR-145
| | | | | | | | | | | | | | |
1749:5′ gaGGAU--GUGAAAAAACUGGAa 3′ PTENP1</td><td>0.8960</td></tr>
<tr><td>3′ ucccuaaGGAC--CCUUUUGACCUg 5′ hsa-miR-145
| | | | | | | | | | | | |
452:5′ guguucaCCUGCUAGCAAACUGGAg 3′ MEG3</td><td>–0.2663</td></tr>
<tr><td>3′ ucccuaaggacccUUUUGACCUg 5′ hsa-miR-145
| | | | | | | |
1744:5′ ggagggaaaaaaaACAACUGGAa 3′ TUG1</td><td>–0.1244</td></tr>
<tr><td>3′ ucccuaaggacCCUUUUGACCUg 5′ hsa-miR-145
| | | | | | | | | | |
1888:5′ aaguuugaaguGGAAAACUGGAa 3′ MALAT1</td><td>–0.0110</td></tr>
<tr><td>3′ uguUUCAAGACAUCACGUGACu 5′ hsa-miR-148a
| | | | | | | | | | | | |
311:5′ acuAAG-UCAUU--UGCACUGg 3′ H19</td><td>–0.9028</td></tr>
<tr><td>3′ uguuucaagACAUCACGUGACu 5′ hsa-miR-148a
| | | | | | | | | |
4474:5′ cuacucgaaUG-AAUGCACUGg 3′ TUG1</td><td>–0.0699</td></tr>
<tr><td>3′ uguuucaagacaucaCGUGACu 5′ hsa-miR-148a
| | | | | |
2033:5′ caagcacuaccaccaGCACUGu 3′ TUG1</td><td>–0.0004</td></tr>
<tr><td>3′ uguuucaagacaucaCGUGACu 5′ hsa-miR-148a
| | | | | |
2203:5′ aaauuggauaaaauaGCACUGa 3′ MALAT1</td><td>–0.0014</td></tr>
<tr><td>3′ ggggagGGUCGGAGGGACGGu 5′ hsa-miR-1207-5p
| : : | | | | | | | | |
47:5′ cgugggCUGGAC-CCCUGCCc 3′ MEG3</td><td>–0.0239</td></tr>
<tr><td>3′ ggggagggucggagGGACGGu 5′ hsa-miR-1207-5p
| | | | | |
865:5′ cucaucucuuaagaCCUGCCg 3′ UCA1</td><td>–0.0155</td></tr>
<tr><td>3′ ggggAGGG-UCGGAGGGACGGu 5′ hsa-miR-1207-5p
| | | : : | : | | | | | | | |
267:5′ cuggUCCUGGGUCACCCUGCCa 3′ MUDENG</td><td>–0.5985</td></tr>
<tr><td>3′ aguuguaGUCAGACUAU-UCGAu 5′ hsa-miR-21
: | | | | | | | | | | | | | |
137:5′ guguuugUAGUCUGAUAGAGCUu 3′ MUDENG</td><td>–0.5634</td></tr>
<tr><td>3′ aguuguagucagacuAUUCGAu 5′ hsa-miR-21
| | | | | |
451:5′ uccaaaucuaaguacUAAGCUc 3′ MUDENG</td><td>–0.0531</td></tr>
<tr><td>3′ agUUGUAGUCAGACUAUUCGAu 5′ hsa-miR-21
| : : : | : | : | | | | | |
559:5′ aaAGUGUUUAAAUAGUAAGCUg 3′ MUDENG</td><td>–0.0866</td></tr>
<tr><td>3′ agUUGUAGUCAGA---CUAUUCGAu 5′ hsa-miR-21
| :   : | : | | | : | : | | | | | |
656:5′ cuAGAGUUAGUUUUUAAGUAAGCUu 3′ MUDENG</td><td>–0.0626</td></tr>
</table>

3′ aguuguagucagacuAUUCGAu 5′ hsa-miR-21<br>| | | | | | 895:5′ guagagaguaucauuUAAGCUu 3′ **MUDENG**	–0.0689
3′ aguUGUAGUCAGACUAUUCGAu 5′ hsa-miR-21<br>| | | | | | | | | | | | | | 3752:5′ gauAAAUGAG-CUAAUAAGCUu 3′ **TUG1**	–0.0828
3′ aguuguagucagacuAUUCGAu 5′ hsa-miR-21<br>| | | | | | 6630:5′ gauccaaguauuuauUAAGCUa 3′ **TUG1**	–0.0019
3′ aguuguagucagacuAUUCGAu 5′ hsa-miR-21<br>| | | | | | 6100:5′ uucuuaagcagagauUAAGCUu 3′ **TUG1**	–0.0017

Supplementary Table 8: mRNAs targeted by miRNA by MiRanda tool

MicroRNA interaction with mRNA	mirSVR score
3′ ucccuaaggacccuuUUGACCUg 5′ hsa-miR-145<br>| | | | | | | 915:5′ gucaacgcaacgucuAACUGGAc 3′ **FGFR2**	–0.2628
3′ ucccuaaggacccuuUUGACCUg 5′ hsa-miR-145<br>: | | | | | | 190:5′ acagaugggguctguGACUGGAu 3′ **KLF4**	–0.0222
3′ ucccUAAGG--ACCCUUUUGACCUg 5′ hsa-miR-145<br>| | | | | | | : : | | | | | | 260:5′ caaaAUGCCAAGGGGGUGACUGGAa 3′ **KLF4**	–0.0520
3′ ucccuaaggacccuuuUGACCUg 5′ hsa-miR-145<br>| | | | | | 209:5′ uuaucuuuucugaugcACUGGAc 3′ **EIF2C4**	–0.0605
3′ ugUUUCAAGACAUCACGUGACu 5′ hsa-miR-148a<br>| | | | | | | | | | | | | | | 643:5′ agACAG-UCUGUUAUGCACUGu 3′ **KLF4**	–1.0910
3′ uguuucaagacaucACGUGACu 5′ hsa-miR-148a<br>| | | | | | | 34:5′ aggaauccccaacaUGCACUGa 3′ **DNMT1**	–1.0394
3′ uguuUCAAGAC--AUC-ACGUGACu 5′ hsa-miR-148a<br>| | | | | | | | | | | | | | | 110:5′ cagcAGCUCGGAAUAGUUGCACUGa 3′ **EIF2C4**	–0.7046
3′ uguuucaagacaucaCGUGACu 5′ hsa-miR-148a<br>| | | | | | 134:5′ aaucuauacuuugcaGCACUGu 3′ **EIF2C4**	–0.0117
3′ uguuucAAGACAUCACGUGACu 5′ hsa-miR-148a<br>| | : | | : | | | | | | | 208:5′ auuaucUUUUCUGAUGCACUGg 3′ **EIF2C4**	–0.8675
3′ uguuucaagacaucaCGUGACu 5′ hsa-miR-148a<br>| | | | | | 1768:5′ ucccagcaauauaaaGCACUGu 3′ **EIF2C4**	–0.0856

<table>
<tr><td>3′ uguuucaagacaucaCGUGACu 5′ hsa-miR-148a
| | | | | |
18:5′ uccccaaacaggacaGCACUGg 3′ FGFR2</td><td>–0.0012</td></tr>
<tr><td>3′ aguUGUAGUCAGA--CUAUUCGAu 5′ hsa-miR-21
| | | : : | : | | | | | | | |
1082:5′ gucACUUUGUUUUAAAAUAAGCUa 3′ EIF2C4</td><td>–1.0566</td></tr>
<tr><td>3′ aguuguagucagacuAUUCGAu 5′ hsa-miR-21
| | | | | |
1912:5′ aggccuguugcucauUAAGCUg 3′ EIF2C4</td><td>–0.0070</td></tr>
</table>

“Good” mirSVR score refers to miRNA targets with <−0.1 score, and “non-good” mirSVR score refers to targets with >−0.1 score obtained from the support vector regression algorithm mirSVR.

Supplementary Table 9: lncRNAs targeted by miRNA from Starbase

<table>
<tr><th>miRName</th><th>LncRNA</th><th>Cancers Num</th><th>Target site</th></tr>
<tr><td>miR-21-5p</td><td>TUG1</td><td>4</td><td>miRNA 3′-agtTGTAGTCAGACTATTCGAt-5′
| | | | | | | | || | | | | |
ncRNA 5′-gatAAATGAG-CTAATAAGCTt-3′</td></tr>
<tr><td>miR-145-5p</td><td>TUG1</td><td>2</td><td>miRNA 3′-tccctaaggacccTTTTGACCTg-5′
| | | | | | | |
ncRNA 5′-ggagggaaaaaaaACAACTGGAa-3′</td></tr>
<tr><td>miR-145-5p</td><td>MALAT1</td><td>2</td><td>miRNA 3′-tccctaaggacCCTTTTGACCTg-5′
| | | | | | | | | ||
ncRNA 5′-aagtttgaagtGGAAAACTGGAa-3′</td></tr>
<tr><td>miR-148a-3p</td><td>H19</td><td>7</td><td>miRNA 3′-tgtTTCAAGACATCACGTGACt-5′
| | | | | | | | | | | | |
ncRNA 5′-actAAG-TCATT--TGCACTGg-3′</td></tr>
<tr><td>miR-148a-3p</td><td>HOTAIR</td><td>3</td><td>miRNA 3′-tgtttcAAGACATCACGTGACt-5′
| | : | | | | | | | | | |
ncRNA 5′-gatgcaTTTTCT-GTGCACTGg-3′</td></tr>
<tr><td>miR-148a-3p</td><td>CCAT1</td><td>N/A</td><td>miRNA 3′-tgtttcaAGACATC-ACGTGACt-5′
| : | | || | | | | | | |
ncRNA 5′-catcgacTTTGAAGTTGCACTGa-3′</td></tr>
</table>

Supplementary Table 10: Validated targeting miRNAs from miRTarBase

Genes	Targeting miRNAs
DNMT1	miR-148a-3p,miR-29b-3p,miR-152-3p,miR-301b-3p,miR-126-3p,miR-342-3p,miR-185-5p,miR-140-5p,miR-193b-3p,miR-148b-3p,miR-155-5p,miR-877-3p,miR-484,miR-149-5p,miR-30b-5p,miR-100-5p,miR-26a-5p,miR-1260b,miR-200a-3p,miR-200b-3p,miR-429,miR-29a-3p,miR-92a-3p,miR-20a-5p,miR-19b-3p,miR-19a-3p,miR-18a-5p,miR-17-5p,let-7c-5p,miR-218-5p
AGO4	miR-21-5p,miR-335-5p,miR-155-5p,miR-484,miR-92a-3p,miR-20a-5p,miR-33a-3p,miR-548a-5p,miR-15a-5p,miR-16-5p,miR-15b-5p,miR-195-5p,miR-424-5p,miR-497-5p,miR-545-3p,miR-3121-3p,miR-6079,miR-6828-5p,miR-6838-5p,miR-137,miR-6071,miR-192-3p,miR-6828-3p,miR-582-5p,miR-671-5p,miR-6782-3p,miR-144-3p,miR-101-3p,miR-6507-5p,miR-3692-5p,miR-93-3p,miR-129-5p,miR-6832-3p,miR-6834-3p,miR-513b-3p
KLF4	miR-145-5p,miR-10b-5p,miR-25-3p,miR-135b-5p,miR-130a-3p,miR-663a,miR-103a-3p,miR-107,miR-335-5p,miR-128-3p,miR-124-3p,miR-30b-5p,miR-29a-3p,miR-15a-5p,miR-624-3p,miR-34a-5p,miR-7-5p,miR-137,miR-152-3p,miR-206
EGFR	miR-145-5p,miR-21-5p,miR-1-3p,miR-16-5p,miR-7-5p,miR-128-3p,miR-146a-5p,miR-542-5p,miR-128b,miR-133a-3p,miR-133b,miR-27a-3p,miR-335-5p,miR-155-5p,miR-30a-5p,let-7a-5p,miR-301b-3p,miR-574-3p,miR-219a-5p,miR-302b-3p,miR-125a-5p,miR-218-5p
FGFR2	miR-19b-1-5p, miR-125b-5p, miR-1-3p, miR-186-5p

miRNAs presented in bold fonts are having common MREs with the target genes and the lncRNAs screened in the present study.

Supplementary Table 11: mRNAs targeted by miRNA by DIANA tools, TargetScan, miRTarBase

NCBI ID	mRNA	miRNA
NM_001130823.2	DNMT1	miR-148a-3p
NM_017629.3	AGO4	miR-21-5p
NM_001314052.1	KLF4	miR-145-5p
NM_005228.3	EGFR	miR-21-5p, miR-145-5p
NM_000141.4	FGFR2	miR-125b-5p

Supplementary Table 12: mRNAs targeted by miRNA from Starbase

NCBI ID	mRNA	miRNA
NM_001130823.2	DNMT1	miR-148a-3p
NM_017629.3	AGO4	N/A
NM_001314052.1	KLF4	miR-148a-3p, miR-148b-3p, miR-145-5p
NM_005228.3	EGFR	miR-148a-3p, miR-148b-3p
NM_000141.4	FGFR2	miR-145-5p

Supplementary Table 13: mRNAs targeted by miRNA from ceRDB

NCBI ID	mRNA	miRNA name
NM_001130823.2	DNMT1	miR-148
NM_017629.3	AGO4 (or)EIF2C4	miR-21, miR-148
NM_001314052.1	KLF4	miR-145, miR-148
NM_005228.3	EGFR	N/A
NM_000141.4	FGFR2	N/A

Supplementary Table 14: miRNA targeting both lncRNA-mRNA from LncACTdb

lncRNA Ensembl ID	lncRNA	miRNA name	gene Ensembl ID	gene name
ENSG00000130600	H19	hsa-miR-148a	ENSG00000130816	DNMT1
ENSG00000253352	TUG1			
ENSG00000251562	MALAT1			
ENSG00000245532	NEAT1			
ENSG00000130600	H19	hsa-let-7a	ENSG00000134698	EIF2C4
ENSG00000214548	MEG3			
ENSG00000253352	TUG1			
ENSG00000251562	MALAT1			
ENSG00000245532	NEAT1			
ENSG00000130600	H19	hsa-miR-145	ENSG00000136826	KLF4
ENSG00000253352	TUG1			
ENSG00000251562	MALAT1			
ENSG00000245532	NEAT1			

ENSG00000130600	H19	hsa-miR-21	ENSG00000146648	EGFR
ENSG00000253352	TUG1			
ENSG00000251562	MALAT1			
N/A	N/A	N/A	N/A	FGFR2

Supplementary Table 15: miRNA targeting both lncRNA-mRNA by miRSponge

Sponge Type	Sponge Name	mRNA	miRNA name	Reference (PMID)
Coding-mRNA	DNMT1	n/a	miR-148a	24714841
Host gene	H19	TGFB1	miR-675	24988946
Host gene	H19	RUNX1/ Smad/cdc6	miR-675	24532688
Pseudogene	PTENP1	PTEN	miR-21	20577206
LncRNA	GAS5	n/a	miR-21	23933812
LncRNA	lincRNA-RoR	OCT4/Nanog/Sox2	miR-145	23541921
LncRNA	lincRNA-RoR	n/a	miR-145	24589415

Supplementary Table 16: Statistical summary of miRNA expression

(a) *P*-values of gastric cancer associated miRNAs between tumor and normal

miRNA	*P* value	*P* value summary
miR-21	0.7875	ns
miR-145	0.1659	ns
miR-148a	0.1537	ns

(b) Association of MRE shared miRNAs expression with tumor clinicopathological features

miRNA	Diffuse Vs. Intestinal	< Stage II Vs. > Stage II	<T2 Vs. >T2	<N2 Vs. >N2	Tobacco user Vs. Non-user	Alcoholic Vs. Non-alcoholic	Veg Vs. Non-Veg
miR-21	0.9425	0.1097	0.2551	0.1463	0.9587	0.7819	0.1685
miR-145	0.2187	0.0171#	0.0245#	0.0455#	0.5867	0.9059	0.5904
miR-148a	0.3311	0.9381	0.4659	0.5801	0.5540	0.0470#	0.9009

[#] *P* value with statistical significance.

Supplementary Table 17: Statistical summary of mRNA expression

(a) *P*-values of gastric cancer associated mRNAs between tumor and normal

mRNA	*P* value	*P* value summary
DNMT1	0.8078	ns
AGO4	0.612	ns
KLF4	0.0803	ns
EGFR	0.1981	ns
FGFR2	0.0449	*

[*]*P* value with statistical significance.

(b) Association of gastric cancer related mRNAs expression with tumor clinicopathological features

	Diffuse Vs. Intestinal	< Stage II Vs. > Stage II	<T2 Vs. >T2	<N2 Vs. >N2	Tobacco user Vs. Non-user	Alcoholic Vs. Non-alcoholic	Veg Vs. Non-Veg
DNMT1	0.2187	0.5174	0.6872	0.9089	0.3181	0.7174	0.5075
AGO4	0.9194	0.9413	0.9090	0.6132	0.5818	0.3517	0.4309
KLF4	0.9425	0.6283	0.7480	0.6132	0.7045	0.8450	0.9432
EGFR	0.6411	0.4173	0.5174	0.8990	0.7604	0.3959	0.8776
FGFR2	0.9999	0.4680	0.4192	0.4578	0.9261	0.5174	0.5621

Supplementary Table 18: LncRNA-mRNA spearman correlation

Spearman correlation *P*-values

Genes	DNMT1	AGO4	KLF4	EGFR	FGFR2
H19	0.4228	0.8181	0.2486	0.4269	0.4119
PTENP1-AS	0.9637	0.2048	0.0258#	0.0088#	0.2839
GAS5	0.5111	0.3187	0.1144	0.5157	0.2849
MEG3	0.6225	0.4536	0.0450#	0.0557	0.2849
TUG1	0.6459	0.3972	0.0506	0.0342#	0.5624
AP5M1	0.4608	0.7623	0.2506	0.2576	0.8859
PANDA	0.9358	0.1806	0.0249#	0.0058#	0.4325
MALAT1	0.6077	0.0497#	0.0025#	0.0043#	0.1313
CCAT1	0.6955	0.4451	0.2785	0.1629	0.5341
LINC312	0.2796	0.3474	0.1592	0.2970	0.8290
NBAT1	0.9581	0.5172	0.1155	0.0509	0.7481
HOTAIR	0.6578	0.1759	0.0347	0.0136	0.7445
ZEB2-AS1	0.9507	0.5545	0.0239	0.0152	0.5656
BC032469	0.4395	0.1759	0.0037#	0.0298#	0.0336#
POU3F3	0.4214	0.3585	0.0221#	0.0196#	0.3106
UCA1	0.5929	0.5419	0.0590	0.0314#	0.5187
NEAT1	0.8436	0.3376	0.3673	0.5357	0.6325
FALEC	0.7534	0.1535	0.0467#	0.0304#	0.4812
LINCROR	0.7534	0.1743	0.0157#	0.0981	0.2437

[#] *P* value with statistical significance.

Supplementary Table 19: List of primers used for lncRNA expression profiling

Genes	Primer sequences
AP5M1 - Fwd	5′ - TCTCACGAACCTCAAAACTTCAT - 3′
CCAT1 - Fwd	5′ - GTGTATCTTAGTTCAACCAAATTGTAATCATCTG - 3′
FALEC - Fwd	5′ - CTGCACTCTACACTAGCAGCCT - 3′
GAS5 - Fwd	5′ - TGCCTGATGGAGTCTCATGGCACA - 3′
H19 - Fwd - Fwd	5′ - AGGGCTTCAGCAGGAGCCCTGG - 3′
HOTAIR - Fwd	5′ - CTTGTGTAGGTTGTGTGTGTGTGGTGG - 3′
LINC00312 - Fwd	5′ - AGTGGCACAGCTCATCTTGAAGT - 3′
LINCROR - Fwd	5′ - GCAGCTGTTACGGGAAACGGTA - 3′
MALAT1 - Fwd	5′ - CTGGTGTTTTGAGAAGCCCTACTGCTG - 3′
MEG3 - Fwd	5′ - GGCGCACCCCACTGTGCTCTC - 3′
NBAT1 - Fwd	5′ - TGCTCCACCCATTTGTAAGCAGA - 3′
NEAT1 - Fwd	5′ - TCTTCTTCCCCTTTACAGCACAAAT - 3′
PANDA - Fwd	5′ - GATCTGTCTGCCTCGGTCTC - 3′
POU3F3 - Fwd	5′ - CAGTTGTCACCTGCAAAGACCTCTG - 3′
PTENP1-AS - Fwd	5′ - TGGCTTCTTGCCAGAGCTGTGCT - 3′
TUG1 - Fwd	5′ - GGCCGAGCGAACATGAAACTTTCAACT - 3′
UCA1 - Fwd	5′ - GCGTTTCATGTGGTTTTCCTTTCCT - 3′
ZEB2-AS1 - Fwd	5′ - TGTGCATGATGAATTTCTGGACTGGA - 3′
BC032469 - Fwd	5′ - CGAGCCGGCAGTGGCAAACC - 3′
GAPDH - Fwd	5′ - GAAGAGGGGAGGGGCCTAGG - 3′
Universal cDNA Reverse primer	5′ - CAGTGCAGGGTCCGAGGTACAGAGC CACCTGGGCAATTTTTTTTTTTVN - 3′
Universal RT Reverse primer - lncRNA	5′ - CAG TGC AGG GTC CGA GGT - 3′

3` Wobble bases: V- [A,C,G], N- [A,C,G,T]

Supplementary Table 20: List of stem loop and forward primers used for miRNA expression analysis

Genes	Primer sequences (5′–3′)
miR-21	Stemloop - GTCGTATCCAGTGCGTCGAGTGACACGCAGAGCCACCTGGGCAATTTGCA CTGGATACGACTCAACA
	Fwd - GCCCGCTAGCTTATCAGACTGATG
miR-145	Stemloop - GTCGTATCCAGTGCGTCGAGTGACACGAGAGCCACCTGGGCAATTTGCAC TGGATACGACAGGGAT
	Fwd - GTCCAGTTTTCCCAGGAATC
miR-148a	Stemloop - GTCGTATCCAGTGCGTCGAGTGACACGAGAGCCACCTGGGCAATTTGCAC TGGATACGACACAAAG
	Fwd - CAGTCAGTGCACTACAGAACT
RNU44	Stemloop – GTCGTATCCAGTGCGTCGAGTGACACGAGAGCCACCTGGGCA ATTTGCACTGGATACGACAGTCAG
	Fwd - GCAAATGCTGACTGAACATGA
Universal RT Reverse primer – miRNA/ snoRNA	TCGTATCCAGTGCGTCGAGT
